# Exploring Extracellular Vesicles Biogenesis in Hypothalamic Cells through a Heavy Isotope Pulse/Trace Proteomic Approach

**DOI:** 10.3390/cells9051320

**Published:** 2020-05-25

**Authors:** Chee Fan Tan, Hui San Teo, Jung Eun Park, Bamaprasad Dutta, Shun Wilford Tse, Melvin Khee-Shing Leow, Walter Wahli, Siu Kwan Sze

**Affiliations:** 1 NTU Institute for Health Technologies, Interdisciplinary Graduate School, Nanyang Technological University, Singapore 637335, Singapore; ctan088@e.ntu.edu.sg; 2 School of Biological Sciences, Nanyang Technological University, Singapore 637551, Singapore; teoh0084@e.ntu.edu.sg (H.S.T.); parkjungeun0409@gmail.com (J.E.P.); bama0001@e.ntu.edu.sg (B.D.); swtse@ntu.edu.sg (S.W.T.); 3 Lee Kong Chian School of Medicine, Nanyang Technological University, Singapore 636921, Singapore; melvin_leow@ntu.edu.sg (M.K.-S.L.); Walter.Wahli@ntu.edu.sg (W.W.); 4 Department of Endocrinology, Tan Tock Seng Hospital, Singapore 308433, Singapore; 5 Cardiovascular and Metabolic Disorder Program, Duke-NUS Medical School, Singapore 169857, Singapore; 6 Center for Integrative Genomics, University of Lausanne, Le Génopode, CH-1015 Lausanne, Switzerland

**Keywords:** extracellular vesicles, extracellular vesicles biogenesis, pulsed-SILAC, hypothalamus, energy homeostasis, cathepsin

## Abstract

Studies have shown that the process of extracellular vesicles (EVs) secretion and lysosome status are linked. When the lysosome is under stress, the cells would secrete more EVs to maintain cellular homeostasis. However, the process that governs lysosomal activity and EVs secretion remains poorly defined and we postulated that certain proteins essential for EVs biogenesis are constantly synthesized and preferentially sorted to the EVs rather than the lysosome. A pulsed stable isotope labelling of amino acids in cell culture (pSILAC) based quantitative proteomics methodology was employed to study the preferential localization of the newly synthesized proteins into the EVs over lysosome in mHypoA 2/28 hypothalamic cell line. Through proteomic analysis, we found numerous newly synthesized lysosomal enzymes—such as the cathepsin proteins—that preferentially localize into the EVs over the lysosome. Chemical inhibition against cathepsin D promoted EVs secretion and a change in the EVs protein composition and therefore indicates its involvement in EVs biogenesis. In conclusion, we applied a heavy isotope pulse/trace proteomic approach to study EVs biogenesis in hypothalamic cells. The results demonstrated the regulation of EVs secretion by the cathepsin proteins that may serve as a potential therapeutic target for a range of neurological disorder associated with energy homeostasis.

## 1. Introduction

Cells are known to secrete extracellular vesicles (EVs) into the extracellular milieu that can be categorized into multivesicular bodies (MVBs)-originated exosomes (30–150 nm), microvesicles (MVs) (100–1000 nm) that shed from the plasma membrane or apoptotic bodies (>1000 nm) from dying cells [1]. In particular, exosomes and MVs are known to promote intercellular communication in numerous physiological and pathological settings through the transfer of materials such as proteins, mRNA and miRNA to the recipient cells [2,3,4]. Recent findings have suggested a link between lysosome status and EVs secretion where inhibition of lysosome activity increases EVs secretion [5,6,7]. As the outcome of these processes are vastly different, some studies have been done to understand lysosome and EVs biogenesis, respectively.

In lysosome-mediated protein degradation, the endosomal sorting complex required for transport (ESCRT) machinery is crucial for the sequestration of ubiquitinated proteins at the endosomal membrane, followed by inward budding of the membrane into intraluminal vesicles (iLVs) for degradation [8]. In contrast, formation of EVs seems to involve only a subset of the ESCRT machinery [9] and non-ubiquitinated proteins can be sorted into the vesicle as well [10]. Other ESCRT-dependent mechanism such as the interaction between the syntenin-1, Alix and syndecan proteins was able to promote the biogenesis of CD63^+^ exosomes [11,12]. Exosome biogenesis can also occur independent of the ESCRT machinery through the conversion of sphingomyelin to ceramide by the neutral type II sphingomyelinase (*n-SMase*) that stimulates negative curvature of the endosomal membrane to form iLVs [13,14]. A tetraspanin-enriched micro-domain has also been suggested to promote exosome biogenesis as various tetraspanin knockout mouse model showed either a reduction in exosome number [15] or change in cargo content [10]. On the other hand, some of the mechanism for MVs biogenesis are distinctive from exosome biogenesis as molecular cargos are transported to the plasma membrane for budding and release [16]. The process of MVs biogenesis was shown to require the action of small GTPase such as ADP-ribosylation factor 6 (*ARF6*) [17] and Ras-related protein *Rab-22A (RAB22A)* [18]. Additionally, formation of MVs requires the activity of acid sphingomyelinase (a-SMase) rather than n-SMase, as illustrated in P2X7-dependent MVs biogenesis in glial cells [19]. Lastly, arrestin domain-containing protein 1-mediated relocation of TSG101 from the endosome to the plasma membrane was demonstrated to facilitate ESCRT-dependent MVs biogenesis [20]. However, the mechanism that regulates the balance between EVs secretion and lysosomal degradation remained poorly elucidated

Due to the secretory nature of EVs, it is likely that certain proteins essential for EVs biogenesis should be constantly synthesized to replace those that were secreted out. We postulated that preferential sorting of actively synthesized proteins into the EVs instead of lysosome may reveal a divergent role of these proteins in promoting EVs biogenesis. While EVs proteome had been previously characterized through stable isotope labelling by amino acids in cell culture (SILAC) methodology [21,22,23], we have adopted a variant of this approach [24] to profile the protein synthesis rate rather than the protein concentration of the EVs, lysosome and total cell lysate proteomes of the mHypoA 2/28 adult mouse hypothalamus cell line. This pulsed SILAC (pSILAC)-based quantitative proteomics strategy will allows us to study the EVs protein synthesis rate at a proteome-wide level that is not well characterized, and such information would be pertinent in unravelling novel mechanism on EVs biogenesis. In this current report, we identify a possible role of newly synthesized cathepsin D on EVs biogenesis in mHypoA 2/28 hypothalamic cells and these results may provide invaluable insight into the regulation of the EVs-lysosome axis and their possible effect on energy homeostasis.

## 2. Materials and Methods

### 2.1. Cell Culture and pSILAC Treatment

mHypoA 2/28 adult mouse hypothalamus cells (*CELLutions* Biosystems, Burlington, ON, Canada) were grown in DMEM containing unlabeled “light” ^12^C_6_, ^14^N_2_-L-lysine (146 mg/L) and ^12^C_6_-L-arginine (84 mg/L) (GE Hyclone, Logan, UT, USA), supplemented with 5% EVs-depleted fetal bovine serum (FBS) (Gibco, Waltham, MA, USA) and 1% penicillin/streptomycin (Nacalai Tesque, Kyoto, Japan) at 37 °C in a 5% CO_2_ humidified incubator. EVs-depleted FBS was obtained through ultracentrifugation at 200,000× *g* for 18 h at 4 °C. For pSILAC treatment, the cells were grown in ‘light media for 24 h and thereafter the cells were washed with PBS twice and incubated in SILAC-DMEM (Cambridge Isotope Laboratories, Tewksbury, MA, USA) which contained 5% dialyzed EVs-depleted FBS (Gibco), 1% penicillin/streptomycin and heavy ^13^C_6_-L-Arginine ^13^C_6_-Hydrochloride (84 mg/L) and ^13^C_6_-L-Lysine ^15^N_2_-hydrochloride (146 mg/L) (Cambridge Isotope Laboratories) for 24 h. The conditioned media was then collected for EVs isolation, while the mHypoA-2/28 cells were harvested for either total cell lysate or lysosome isolation. The mHypoA-2/28 cell line was tested negative for mycoplasma contamination [25].

### 2.2. EVs Isolation

The EVs isolation protocol was based on our previous published article [26]. For each EVs isolation, 200 mL of conditioned media was collected from twenty 100 mm culture dishes with a total of 60 million cells approximately, unless otherwise stated. The conditioned media was centrifuged at 2000× *g* for 20 min at 4 °C to remove cellular debris. The supernatant was then concentrated with a VivaSpin 20 centrifugal concentrator, 300 kDa MWCO (Sartorius AG, Goettingen, Germany) at 4000× *g* at 4 °C. The concentrated media were then washed with PBS thrice through the MWCO to remove any non-EVs materials. Thereafter, the concentrated media was centrifuged at 16,000× *g* for 30 min at 4 °C to remove the larger vesicles. The supernatant was collected and diluted in 3 mL PBS to reduce the viscosity. The diluted supernatant was ultra-centrifuged at 100,000× *g* for 16 h at 4 °C, in a Ti55 rotor (Beckman Coulter, Brea, CA, USA). The EVs pellet was reconstituted in PBS containing protease inhibitor for immediate usage or was stored at −20 °C.

### 2.3. Lysosome Enrichment

Lysosome isolation was performed using the lysosome enrichment kit (Thermo Fisher Scientific, Waltham, MA, USA), as per manufacturer protocol. Briefly, the mHypoA-2/28 cell pellet was re-suspended in 800 µL buffer A and incubated on ice for 2 min. The cells were then lysed through probe sonication and mixed with 800 µL Buffer B. The sample was centrifuged at 500× *g* for 10 min at 4 °C to remove cellular debris. 1500 µL of the sample lysate was then added to 500 µL of Optiprep cell separation media (60%). This solution was overlaid onto a discontinuous Optiprep density gradient (17%, 20%, 23%, 27% and 30%) and ultra-centrifuged at 145,000× *g* for 2 h at 4 °C. Five equal volumes were collected, washed, and stored in PBS with protease inhibitor at −20 °C till further usage.

### 2.4. In-solution Digestion and HPLC Fractionation

mHypoA 2/28 cells were lysed with 8M Urea in 100 mM ammonium bicarbonate (ABB), pH 8, supplemented with protease inhibitor. Protein concentration was measured by Bradford assay. 200 µg of protein lysate was reduced in 20 mM dithiothreitol (DTT) for 1 h at room temperature and followed by alkylation with 55 mM iodoacetamide (IAA) in the dark for 45 min, at room temperature. The proteins were then digested with sequencing grade modified trypsin (1:50), (Promega, Madison, WI, USA) overnight at 37 °C and the reaction was subsequently quenched with 0.5% acetic acid (MilliporeSigma, Burlington, MA, USA). The peptides were desalted using the Sep-Pak C18 1cc Vac Cartridge 50 mg (Waters Corp, Milford, MA, USA) and dried with the Eppendorf Concentrator plus (Eppendorf, Hamburg, Germany). Subsequently, the dried peptides were reconstituted with 0.02% NH_4_OH in HPLC water for fractionation. High pH reversed phase high performance liquid chromatography (RP-HPLC) was performed using the Prominence^TM^ HPLC system (Shimadzu, Kyoto, Japan), with the XBridge^TM^ BEH C18 column (130 Å pore size, 4.6 × 250 mm, 5 µm particle size). The mobile phase was comprised of 0.02% NH_4_OH in HPLC water (A) and 0.02% NH_4_OH in 80% acetonitrile (ACN) (B). A 60 min HPLC gradient consisting of 5 min of 3–10% (B), 40 min of 10–35% (B), 5 min of 35–70% (B) and 70–100% (B) was established for peptide separation. 60 fractions were collected and combined into 16 fractions in a concatenated manner. The fractionated samples were dried and stored in −20 °C prior to LC-MS/MS analysis.

### 2.5. In Gel Digestion

EVs and lysosomal proteins were extracted in reducing Laemmli sample buffer. 200 µg of protein samples were resolved in 12% SDS-PAGE at 120 V for 90 min. Protein bands were visualized through staining with 0.2% Coomassie blue solution. For each gel sample, the gel lane was cut into five equal parts and the gel fractions were further diced into 1 mm^2^ gel pieces. The gel pieces were washed in alternating buffer consisting of either 100 mM ABB or 50% ACN in 100 mM ABB to remove impurities. The gel pieces were then dehydrated with 100% ACN and dried in the vacuum concentrator. The proteins were then reduced in 10 mM of DTT at 60 °C for 1 h, followed by alkylation with 55 mM of IAA at room temperature for 45 min, in the dark. The gel pieces were then subjected to washes again to remove excess DTT and IAA. After dehydrating with 100% ACN and drying in the vacuum concentrator, sequencing-grade modified trypsin was added to the gel pieces and protein digestion was performed at 37 °C, overnight. The digested peptides were extracted with 50% ACN and 5% acetic acid, dried in the vacuum concentrator and stored in −20 °C prior to LC-MS/MS analysis.

### 2.6. LC-MS/MS Analysis

The peptides were reconstituted in 0.1% formic acid (FA) in 3% ACN for LC-MS/MS analysis in the Q-Exactive Hybrid Quadrupole-Orbitrap mass spectrometer, coupled with the UltiMate^TM^ 3000 RSLCnano System (Thermo Scientific). For each analysis, 2 µg of sample was injected to the system. The peptides were first concentrated with a Nano-Trap Columns 75–100 µm I.D. x 2 cm (Thermo Fisher Scientific ) and then separated on a Dionex EASY-Spray 75 μm × 10 cm column packed with PepMap C18, 3 μm, 100 Å (Thermo Fisher Scientific). The mobile phase buffers used were 0.1% formic acid (A) and 0.1% formic acid in ACN (B) and a 60 min gradient was used for peptide separation.

The samples were ionized and injected into the Q-Exactive mass spectrometer with an EASY nanospray source (Thermo Fisher Scientific) at an electrospray potential of 1.5 kV. A full MS scan (350–1600 m/z range) was acquired at a resolution of 70,000, with a maximum ion accumulation time of 100 ms. Dynamic exclusion was set as 30 s. The HCD spectral resolution was set to 35,000. Automatic gain control (AGC) settings of the full MS scan and the MS2 scan were 3 × 10^6^ and 2 × 10^5^, respectively. The top 10 most intense ions above the 5000-count threshold were selected for fragmentation in higher-energy collisional dissociation (HCD), with a maximum ion accumulation time of 120 ms. Isolation width of 2 was used for MS2. Single and unassigned charged ions were excluded from MS/MS. For HCD, the normalized collision energy was set to 28% and the underfill ratio was defined as 0.3%.

### 2.7. Database Search

Raw data generated from two biological replicates for each sample were analyzed using the Proteome Discoverer (PD) 2.2 software (Thermo Fisher Scientific). Protein identification was done by mapping against the UniProt Knowledgebase (UniProtKB) for mouse proteins (downloaded on 16 Mar 2017, 87,463 sequences and 38,788,886 residues), using the SequestHT and Mascot search engine. The Proteome Discoverer’s workflow included an automatic target-decoy search tactic along with the Percolator to score peptide spectral matches from both Mascot and SequestHT searches to estimate the false discovery rate (FDR). The Percolator parameters were set to maximum delta Cn = 0.05; target FDR (strict) = 0.01; target FDR (relaxed) = 0.05, validation based on q-value [27].

For SILAC quantitation, the Spectrum Files RC node was used for spectrum recalibration and the peak feature detection setting in the Minora Feature Detector was set as Minimum Trace Length: 5, Minimum number of isotopic peaks: 2 and Maximum ΔRT of Isotope Pattern Multiplets: 0.2 min. Feature mapper was set to True for retention time alignment, with a maximum retention time shift allowed of 10 min and Precursor abundance was based on intensity. The search parameters also included full trypsin digestion with a maximum of two missed cleavage and precursor mass tolerance and fragment mass tolerance were set at 10 ppm and 0.02 Da, respectively. Carbamidomethylation (+57.02) at cysteine was set as fixed modification, oxidation (+15.99) at methionine, deamidation (+0.98) at asparagine and glutamine and heavy ^13^C_6_-L-Arginine ^13^C_6_-Hydrochloride (+6.02) and ^13^C_6_-L-Lysine ^15^N_2_-hydrochloride (+8.01) were set as dynamic modifications. Acetylation (+42.01) at protein N-terminus was set as dynamic modification too. Maximum dynamic modifications were set at 4.

Quantification of the H/L (heavy isotope/light isotope) ratio for each protein is based on precursor ion quantification. The protein abundance was determined by the MS signal intensities of the heavy isotope labelled protein over its unlabeled counterpart. With the default setting, PD 2.2 uses the largest chromatographic peak and quantified the heights of this peak at the apex rather than the integrated peak area for quantification. The ratio calculation was set as Pairwise ratio based and the *p*-value was calculated by ANOVA (background based). The maximum fold change allowed was set to 100. The reported H/L ratio has a range of 0.01 to 100, in which 0.01 represent proteins with only non-labelled peptides and 100 represent protein that either contained only labelled peptides or protein with labelled peptides that has abundance value of more than 100 times of its non-labelled counterpart. The datasets were manually filtered by selecting for proteins with q-value <0.05 and identified in both biological replicates. Gene ontology analysis was performed using the DAVID Bioinformatics v6.8 [28]. Pearson correlation coefficient analysis between the proteomics biological replicates was computed with the R-package; “ggplots2” [29].

### 2.8. Western Blot Analysis

Protein concentration was quantified using the bicinchoninic acid (BCA) or Bradford assay. Thereafter, Laemmli sample buffer was added to the protein samples and boiled at 95 °C for 10 min. The protein samples were resolved in a 12% SDS-PAGE and transferred onto a 0.22 µm PVDF membrane. Subsequently, the membrane was blocked in 5% skimmed milk in TBST and then probed with primary antibodies overnight at 4 °C. The antibodies used included ALIX (#2171), GM130 (#12480), LAMP1 (#9091), EEA1 (#3288), RAB7 (#9367) and RAB11 (#5589) from Cell Signaling Technologies (Danvers, MA, USA), CD9 (sc-18869), cathepsin D (sc-377124), cathepsin L (sc-390367) and VDAC1 (sc-58649) from Santa Cruz Biotechnology (Dallas, TX, USA) and cathepsin B (ab214428), CD63 (ab217345) and CD81 (ab109201) from Abcam (Cambridge, UK).

### 2.9. Nanoparticle Tracking Analysis

The particle number and size distribution of EVs collected from mHypoA 2/28 cells were characterized with the Nanosight NS300 (Malvern Panalytical, Worcestershire, UK) equipped with a 488 nm blue laser and a sCMOS camera. The samples were diluted 200-fold with PBS for analysis using the default protocol as per the manufacturer’s software guide (NanoSight NS300 User Manual). The instrument parameters were set as follow: camera level 5, slider shutter 100, slider gain 200, FPS 32.5, temperature 24 °C, viscosity 0.906–0.910 cP, syringe pump speed 100, capture time 60 s and detect threshold 3. Calculation of particles quantity and size distribution were based on 3 biological replicates.

### 2.10. Cathepsin Inhibition Assay

mHypoA 2/28 cells were seeded onto 100 mm dish and upon reaching 80% confluency, the cells were washed with PBS twice, and treated with 10 µM CA-074Me, C5857 (Sigma Aldrich, St. Louis, MO, USA), 10 µM cathepsin L Inhibitor II (Santa Cruz Technologies) or 20 µM of pepstatin A (Santa Cruz Technologies) in 1% exosome-depleted FBS-DMEM for 24 h and the conditioned media was collected EVs isolation.

### 2.11. Proteinase K Assay

Fifty µg of EVs was supplemented with 5 mM CaCl2 and incubated in either PBS (control), 10 µg/mL of Proteinase K, 1% Triton X-100 or 10 µg/mL of Proteinase K with 1% Triton X-100 for 1 h at 37 °C. The treatment was stopped with the addition of Laemmli buffer supplemented with β-mercaptoethanol and the sample was heated at 95 °C for 15 min prior to immunoblotting.

### 2.12. RNA Isolation and RT PCR

Total RNA was isolated using the NucleoSpin^®^ RNA II kit (Macherey-Nagel GmbH, Duren, Germany). RNA concentration was determined with the NanoDrop 2000 spectrophotometer (Thermo Fisher Scientific). First-strand cDNA was synthesized using the RevertAid RT Reverse Transcription kit (Thermo Fisher Scientific) as per manufacturer’s protocol. Quantitative PCR (qPCR) reactions were performed using the CFX Connect™ Real-Time PCR Detection System (Bio-Rad Laboratories, Inc., Hercules, CA, USA) with the KAPA SYBR^®^ FAST qPCR kit (KAPA Biosystems, Wilmington, MA, USA) under the following condition; initial denaturation at 95 °C for 3 min, followed by 40 cycle of denaturation at 95 °C for 10 s, primer annealing at 60 °C for 30 s and final extension at 72 °C for 30 s.

Each sample was performed in triplicate and relative quantification was determined using the ∆∆CT method. The primer sequences were *RAB11* forward 5′-GAGCTTTTGCAGAGAAGAATGGT-3′, *RAB11* reverse 5′-TTCTGACAGCACTGCACCTT-3′; *RAB27A* forward 5′-AGAGAGTGGTGTACAGAGCCA-3′, *RAB27A* reverse 5′-TTTCACAGTACGCGTGCATC-3′; *RAB27B* forward 5′-TTGGGACACTGCTGGACAAG-3′, RAB27B reverse 5′-TGCCTGCAGTTGACTCATCC-3′; *RAB35* forward 5′-CGTCAATGTGGAAGAGATGTTCA-3′, *RAB35* reverse 5′- GCAGCAGCGTTTCTTTCGTT-3′.

### 2.13. Transmission Electron Microscopy

mHypoA 2/28-derived EVs were diluted 20-fold in PBS and 7 µL of the diluted sample was added onto a glow discharged carbon-coated grid and incubated for 1 min. Thereafter, 2% uranyl acetate was added to the sample and incubated for 1 min. Excess uranyl acetate was blotted off with filter paper. The grid was air-dried for 10 min and subsequently imaged using the T12 Icorr transmission electron microscopy (TEM) at 120 kV (FEI Company, Hillsboro, OR, USA).

## 3. Results

### 3.1. Mass Spectrometric Identification and Quantification of Newly Synthesized Proteins in the Sub-proteome of mHypoA 2/28 Adult Hypothalamus Cell

**Figure 1:**
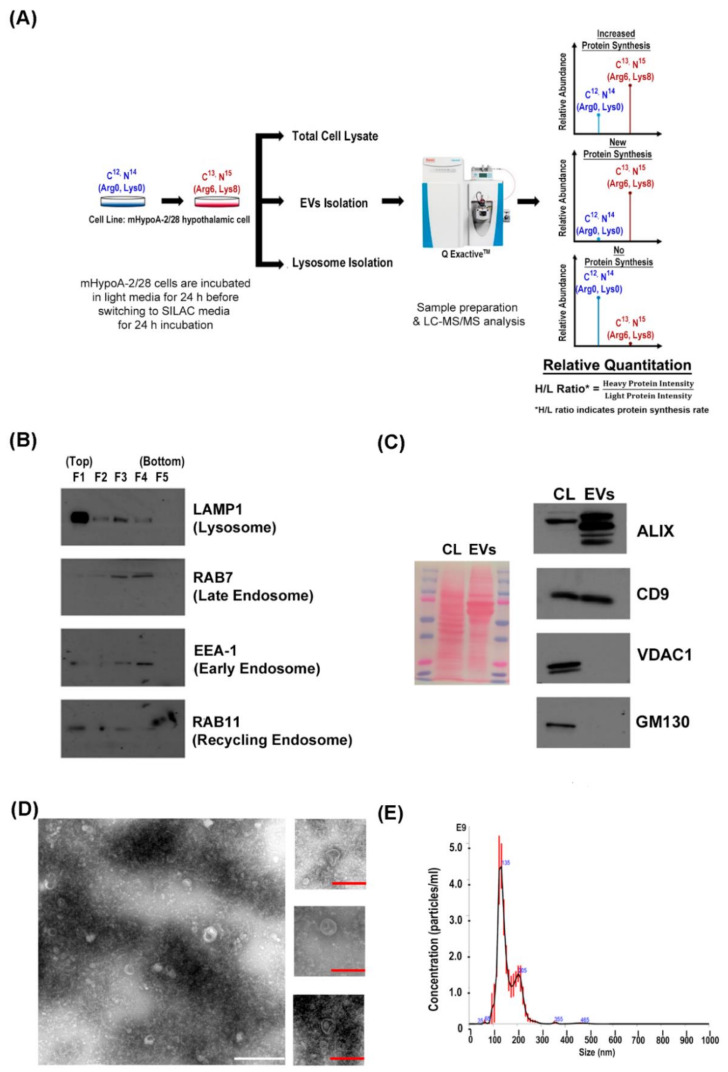
Characterization of lysosomes and EVs isolated from mHypoA 2/28 cells for pSILAC-based quantitative proteomics. (**A**) Schematic diagram illustrating the workflow of pSILAC-based quantitative proteomics analysis of EVs biogenesis through H/L ratio analysis. (**B**) Lysosome isolation was performed using density gradient centrifugation and five fractions were obtained. Equal volume from each fraction were loaded for immunoblotting analysis of lysosomal (LAMP1) and endosomal [EEA-1 (early), RAB7 (late), RAB11 (recycling)] protein markers. (**C**) 50 µg of cell lysate (CL) and EVs proteins were used to probe for known exosomal markers (ALIX and CD9), mitochondrial marker (VDAC) and golgi marker (GM130). Ponceau S staining showed equal loading of proteins for analysis. (**D**) Wide field and closed up TEM images obtained from negative staining of mHypoA 2/28 EVs. The white scale bar represents 500 nm and the red scale bar represents 200nm. (**E**) Nanoparticle tracking analysis of mHypoA 2/28 derived EVs a mean diameter of 158.8 ± 1.3 nm.

A pSILAC-based quantitative proteomics methodology was utilized to profile the proteome of EVs, lysosome and total cell lysate from the mHypoA 2/28 adult hypothalamus cell line for the identification of novel newly synthesized proteins that are enriched in the EVs over the lysosome. We postulated that these newly synthesized proteins are important for EVs biogenesis and will further our understanding in the regulation between EVs secretion and lysosomal activities. As illustrated in Figure 1A, the adult hypothalamus cell line, mHypoA 2/28, was first grown in light DMEM that contained unlabeled L-lysine and L-arginine (light) and subsequently exposed to heavy DMEM that contained stable isotope-labelled L-lysine and L-arginine (heavy) for a period of 24 h. The conditioned media was collected for EVs enrichment while the labelled cells were harvested for lysosome isolation and total protein lysate. The samples were then analyzed using high resolution and high mass accuracy LC-MS/MS for the identification of light and heavy labelled proteins.

To begin with, crude cell lysate was separated by ultracentrifugation on a discontinuous Optiprep density gradient for lysosome isolation and immunoblotting analysis of LAMP-1 protein indicated that majority of the lysosomes were isolated at the top fraction (F1, Figure 1B) while EEA-1 and RAB7 protein expression analysis indicated that the early and late endosomes were found at the lower fractions (F3 and F4, Figure 1B). Subsequently, the F1 fraction was subjected to mass spectrometry analysis of lysosome content. EVs were isolated from conditioned media through a series of ultrafiltration and ultracentrifugation steps. The presence of exosome-like vesicles was confirmed by the detection of known exosomal markers such as ALIX and CD9 and there was minimal cellular contamination as indicated by the absence of VDAC1 (mitochondrial marker) and GM130 (golgi marker) proteins in the EVs preparation (Figure 1C). Transmission electron microscopy (TEM) imaging of isolated EVs revealed a cup-shaped morphology that is typical of TEM-imaged exosome due to processing artifact [30]. The size of these vesicles was generally below 200 nm (Figure 1D). Nanoparticle tracking analysis (NTA) measurement further confirmed that majority of the isolated particles to be within 200 nm and have a mean size of 158.8 nm, which are the typical size for EVs isolated via ultracentrifugation [31] (Figure 1E). Furthermore, 24 of the top 25 common exosome proteins in the Exocarta [32] as well as 73 of the top 100 EVs proteins in Vesiclepedia [33] were identified in our EVs proteome dataset, which further confirmed the enrichment of EVs during sample preparation (Figure S1).

Raw data files generated from 2 biological replicates (three injections) per sample were processed using the Proteome Discoverer software v2.2, with the Mascot and SequestHT search engine. 545, 1545 and 7327 proteins were identified in the EVs, lysosome and total cell lysate samples respectively (FDR at peptide level <1%) (Figure S2A). A cut-off value of H/L ratio >1.5 was used to denote proteins with increased protein synthesis rate. In the cell lysate sample, 3037 proteins have a H/L ratio <1.5 and 3025 proteins have a H/L ratio >1.5, while in the lysosome sample, 620 proteins have a H/L ratio <1.5 and 663 proteins have a H/L ratio >1.5 and lastly, the EVs contained 301 proteins that have a H/L ratio <1.5 and only 90 proteins that have a H/L ratio >1.5 (Figure S2B). Although this data suggests that only 23% of the EVs proteome had increased synthesis rate, this percentage might have been skewed lower by possible serum contaminants found within the EVs proteome. 68 proteins were identified as possible serum contaminants as they had H/L ratio of 0.01 in the EVs proteome and were absent in both the cell lysate and lysosome proteome, based on cluster analysis. Subsequently, only quantified proteins that were detected in both the biological replicates were considered for further analysis, unless otherwise indicated. Pearson’s correlation coefficient between the two biological replicates of the EVs, lysosome and cell lysate data were 0.896, 0.900 and 0.868 respectively, indicating high reproducibility from the proteome dataset (Figure S2C–E).

### 3.2. Extracellular Vesicles Biogenesis is an Active Process that Required Newly Synthesized Protein

One of the main postulation in this study was whether the proteins in the EVs cargo originated from existing cytosolic protein or was it a regulated process in which the proteins were actively synthesized by the cells and sorted into the EVs to promote EVs biogenesis and secretion. To evaluate this hypothesis, gene ontology analysis was carried out on the three-proteome dataset. Based on their H/L ratio (actively synthesized protein: H/L ratio >1.5; slow turnover protein: H/L ratio <1.5), the respective proteome dataset were functionally annotated using the DAVID Bioinformatics tool [28] to elucidate the relevant biological processes associated with EVs biogenesis. In particular, the analysis revealed that a subset of newly synthesized proteins from the cell lysate and EVs proteome promoted the positive regulation of exosome secretion (Table 1). In the cell lysate proteome (Table 1A), these newly synthesized proteins included the vacuolar protein sorting-associated protein 4A (VPS4a), vacuolar protein sorting-associated protein 4B (VPS4b) and hepatocyte growth factor-regulated tyrosine kinase substrate (HGS) that are part of the ESCRT machinery involved in MVBs formation [8,34]; ALIX (PDCD6IP), syndecan-1 (SDC1) and syntenin-1 (SDCBP) proteins that are involved in heparanase-mediated exosome biogenesis [11,12] and Ras-related protein RAB-7a (RAB7A) that is involved in exosome release [11,35]. Similarly, the EVs proteome (Table 1B) contained newly synthesized ALIX, RAB7A and syntenin-1 proteins.

Further H/L ratio analyses were done on proteins involved in known lysosomal and EVs biogenesis pathway to determine if these processes require active protein synthesis to support their function. The data suggested the ESCRT machinery that support exosome formation and lysosomal degradation required active protein synthesis. 28 of the 31 ESCRT-related proteins were identified in the mHypoA 2/28 cell lysate, of which 22 of these proteins showed increased protein synthesis (H/L ratio > 0.6 [log transformed]) (Table 2). Next, mHypoA 2/28 EVs were enriched in proteins from the ESCRT-I complex (Table 2A) as compared to the other ESCRT complexes (Table 2B–E), with TSG101 and VPS28 showing increased protein synthesis. The EVs also contained newly synthesized CHMP4B (ESCRT-III) and ALIX. Taken together, this indicated that only a subset of ESCRT-related proteins is packaged into the EVs during EVs biogenesis. The Bro1 domain in ALIX binds to CHMP4B [36] while the C-terminus of ALIX contained a proline-rich region that binds to N-terminus UBC-like and proline-rich domain of TSG101 [37]. This implied that the ALIX-mediated association of the ESCRT-1 and ESCRT-III complex is packaged into the EVs during membrane scission [38] and hence the proteins have to be constantly synthesized to maintain the biogenesis and function of the EVs.

Lately, alternative mechanisms for MVBs formation were reported. The syntenin-syndecan-ALIX complex [11] was recently proposed as an ESCRT-dependent mechanism that facilitate exosome biogenesis. Heparanase activity in the endosome promotes the trimming of the heparan sulphate group on the syndecan proteins which in turn facilitates the clustering and sorting of ALIX-ESCRT complex into the iLVs via syntenin-1 adaptor protein [12]. The dataset revealed that both ALIX and syntenin-1 to have increased synthesis in the EVs (ALIX: 5.64, syntenin-1: 2.72) and lysosome (ALIX: 1.93, syntenin-1: 10.80) fractions (Table 3A). The syndecan-1 protein is usually cleaved into syndecan-1 C-terminal fragment when inserted into the exosome [12], the truncated form of syndecan-1 may be the reason why it was not detected in the EVs proteome dataset. Nonetheless, cellular syndecan-1 has an H/L ratio of 100 which indicated that syndecan-1 underwent active protein synthesis to replenish the cleaved syndecan-1 that was supporting exosome biogenesis.

Next, the tetraspanin proteins (CD9, CD63 and CD81) were also shown to promote ESCRT-dependent and -independent MVBs formation [10,39,40,41]. Analysis of their protein synthesis ratio revealed that both CD9 and CD81 were actively synthesized in the ribosome and sorted into the EVs cargo (CD9: 2.40, CD81: 10.34) and lysosome (CD9: 3.41, CD81: 30.61) fractions whereas CD63 had a lower synthesis rate (CD63 in EVs: 0.19, CD63 in lysosome: 0.73) (Table 3B). As CD63 is enriched at the MVBs while CD9 and CD81 are localized at the plasma membrane [31], this suggested that plasma membrane-associated EVs (CD9/CD81) may have a higher secretion rate as compared to MVBs-associated EVs (CD63). In support of this view, a recent study demonstrated that both CD9^+^ and CD81^+^ EVs displayed a much higher budding rate than CD63^+^ EVs [42].

Lastly, both acid and neutral sphingomyelinases were reported to promote EVs biogenesis and secretion through the enzymatic cleavage of sphingomyelin into ceramide at its optimal pH [14,19].The mHypoA-2/28 cells contained all three sphingomyelinase, SMPD1, 2 and 3 (Table 3C). SMPD1 was found in the EVs fraction and SMPD3 was found in the lysosome fraction respectively (Table 3C). Taken together, initial pSILAC-based quantitative proteomics analysis established that proteins involved in EVs biogenesis are actively synthesized to maintain its function.

### 3.3. Hierarchical Clustering Analysis Revealed the Preferential Localization of Newly Synthesized Cathepsin Proteins into the EVs

**Figure 2:**
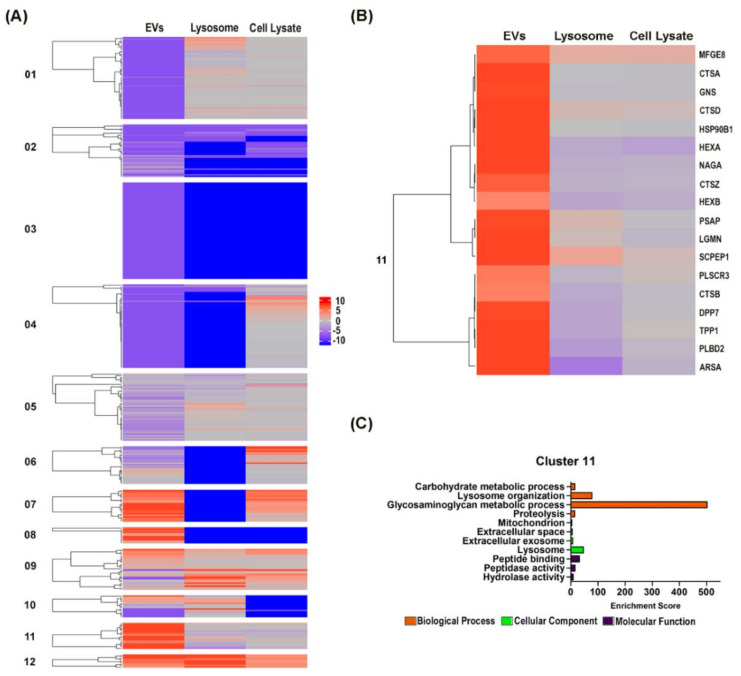
Hierarchical clustering analysis revealed preferential localization of actively synthesized lysosomal-associated proteins in the mHypoA 2/28 EVs. (**A**) Heatmap of the log2 fold change of the mHypoA 2/28 EVs proteome against their counterpart proteins in the lysosome and total cell lysate dataset was plotted with Complex Heatmap [43]. (**B**) Cluster 11 contained 18 proteins that had actively synthesized proteins preferentially localized to the EVs. (**C**) Gene ontology analysis of proteins from Cluster 11. Orange bar represents the biological process, green bar specifies the cellular component and purple bar denotes the molecular function (Enrichment score: *p*-value <0.05).

We postulated that the preferential localization of newly synthesized proteins into the EVs rather than the lysosome and total cell lysate may indicate their divergent role toward EVs biogenesis. To identify these proteins, hierarchical clustering analysis was applied on the three-proteome dataset. Clustering analysis was done based on proteins identified in the EVs proteome and together with the corresponding proteins in the other two datasets and 12 clusters were identified with differential expression profile (Figure 2A). 18 proteins were identified in cluster 11 (Figure 2B) that had newly synthesized proteins preferentially localized to the EVs rather than the lysosome and cell lysate. Most of these proteins are localized to the extracellular exosome (GO: 0070062) (17 proteins) and lysosome (GO: 0005764) (14 proteins) (Figure 2C) and they are mostly lysosome-associated enzymes that functioned in hydrolase activity (GO: 0016787), peptide binding (GO:0042277) and peptidase activity (GO: 0008233). They are also involved in biological processes such as proteolysis (GO: 0006508), glycosaminoglycan metabolic process (GO:0030203), lysosome organization (GO:0007040) and carbohydrate metabolic process (GO:0005975). Although these proteins are found in both EVs and lysosome, the preferential enrichment of these newly synthesized proteins in only the EVs fraction suggested their prospective role in EVs biogenesis and further investigation into these proteins may unravel novel mechanism that helps in delineating EVs biogenesis from lysosome degradation pathway.

**Figure 3:**
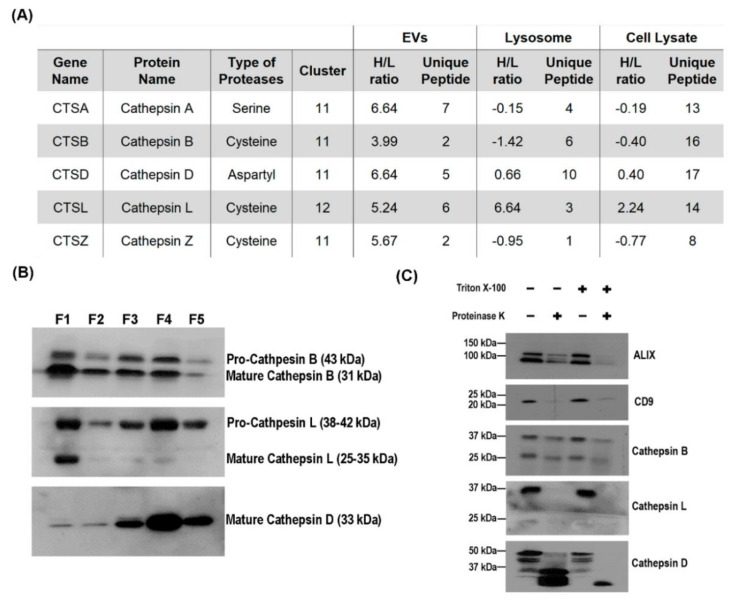
Actively synthesized cathepsin proteins are sorted into mHypoA 2/28 EVs. (**A**) List of cathepsin proteins identified in the three proteomic datasets. (**B**) Location of CTSB, CTSD and CTSL along the endo-lysosomal compartment. mHypoA 2/28 cell lysate was separated on a density gradient (based on Figure 1B) and five fractions were collected. 20 µl from each fraction was used for immunoblotting analysis. (**C**) Proteinase K protection assay was performed to determine the distribution of cathepsin proteins on the EVs. Intact mHypoA 2/28-derived EVs were incubated in either PBS, 10 µg/mL of Proteinase K, 1% Triton X-100 or 10 µg/mL of proteinase K with 1% Triton X-100 for 1 h at 37 °C. Immunoblotting analysis revealed the localization of CTSB, CTSD and CTSL on the EVs. ALIX is a marker for luminal EVs protein while CD9 is a membrane bound EVs protein.

Several cathepsin proteins such as cathepsin A (CTSA), cathepsin B (CTSB) cathepsin D (CTSDCTSD) and cathepsin Z (CTSD) were identified in cluster 11 while cathepsin L (CTSL), which had a different expression profile, was identified and sorted to cluster 12 (Figure 3A). The cathepsin proteins are lysosomal proteinases that function mainly towards protein degradation and recycling and they are classified based on the key catalytic group within its active site [44,45]. On the other hand, cathepsin proteins have also been identified in EVs samples as well [26,46]. Functional role of EVs cathepsin was previously established when presence of CTSB in R3/1 exosomes, stimulated by oxidative stress, promoted RAGE protein expression in recipient cells that is associated with pulmonary fibrosis [47]. Furthermore, elevated level of CTSD expression in neural-derived plasma exosome has been identified in Alzheimer’s patients [48]. Taken together, newly synthesized cathepsins that get preferentially sorted into the EVs may have a functional role in EVs biology.

Interestingly, our dataset revealed that the EVs contain only newly synthesized CTSD while there is a mixture of newly synthesized and pre-existing CTSD proteins in the lysosome and cell lysate (Figure 3A). On the other hand, lysosome-derived CTSL proteins are newly synthesized whereas the EVs and cell lysate contained a mixture of newly synthesized and pre-existing CTSL. The preferential enrichment of these newly synthesized cathepsin proteins into the respective organelle suggest their differential functions toward EVs biogenesis and lysosomal degradation. Further investigation was done to elucidate the role of CTSB, CTSD and CTSL in EVs biogenesis. The intracellular localization of the cathepsin proteins were determined through immunoblotting analysis of organelles obtained from the lysosomal separation density gradient that was established in Figure 1B. Both mature and pro-CTSB were found in all five fractions with mature CTSB having the highest expression in F1 (corresponding to the lysosomal fraction) (Figure 3B). Pro-CTSL was also found in all fraction but mature CTSL was particularly enriched in F1. Lastly, only mature CTSD was identified in the gradient and it was enriched in F3 and F4 which corresponded to the early and late endosome fractions. While the lysosome is thought to be the terminal storage site for acid hydrolase, certain cathepsins such as CTSD was shown to accumulate in the endosome which correlate with the finding [49]. Moreover, mature CTSD found in the endosome are known to mediate proteolytic activities, indicating that they are not just transiently transported across the endosome compartment [50].

Next, to investigate the localization of the cathepsin proteins within the EVs, proteinase K protection assay was conducted on intact mHypoA 2/28 derived EVs (Figure 3C). The EVs membrane protects EVs luminal proteins against proteinase K digestion and this protection is lost when the membrane is solubilized by detergent prior to enzyme digestion. Membranous EVs proteins are digested by proteinase K even in the absence of detergent. ALIX was used as the control for EVs lumen protein while CD9 as the control for EVs membrane protein. The digestion profile confirmed that both CTSB and CTSD are EVs luminal proteins as they had similar expression profile as ALIX.

On the other hand, only pro-CTSL was identified in the EVs and similar to the CD9 protein, pro-CTSL was sensitive to proteinase K digestion even in the absence of Triton X-100, indicating that it is an EVs membrane protein. The assortment of M6PR dependent and independent mechanism for sorting cathepsins into the endosomal compartment may explain the differential localization of CTSB, CTSD and pro-CTSL in the EVs. The type 1 transmembrane protein SEZ6L2 was identified as a M6PR-independent receptor that facilitated CTSD transportation to the endosome [51]. Another mechanism involving the LDL receptor and LDL receptor-related protein 1 (Lrp1) was established in the transportation of non-phosphorylated CTSB and CTSD to the lysosome in a secretion-recapture manner [52]. On the other hand, pro-CTSL was found to co-localize with CD63 in MVBs and could self-aggregate at the membrane of dense vesicle for secretion [53,54]. Taken together, the preferential enrichment of CTSD in the EVs as well as their enrichment in the endosome indicated a differential role of CTSD from CTSB and CTSL in EVs biogenesis.

### 3.4. Chemical Inhibition of Cathepsin D Promote EVs Secretion and Alter EVs Content

Small molecule inhibitors against cathepsin proteolytic activities have been widely used to understand their various functions in physiological and pathological settings [55]. Here, three different inhibitors were utilized to study the role of CTSB, CTSD and CTSL in EVs biogenesis. The inhibition study was conducted by treating mHypoA 2/28 with media containing 10 µM of CA-074 Me, 10 µM of CTSL-i or 20 µM of Pep-A for 24 h and thereafter both cell lysate and conditioned media were collected for further analysis.

**Figure 4:**
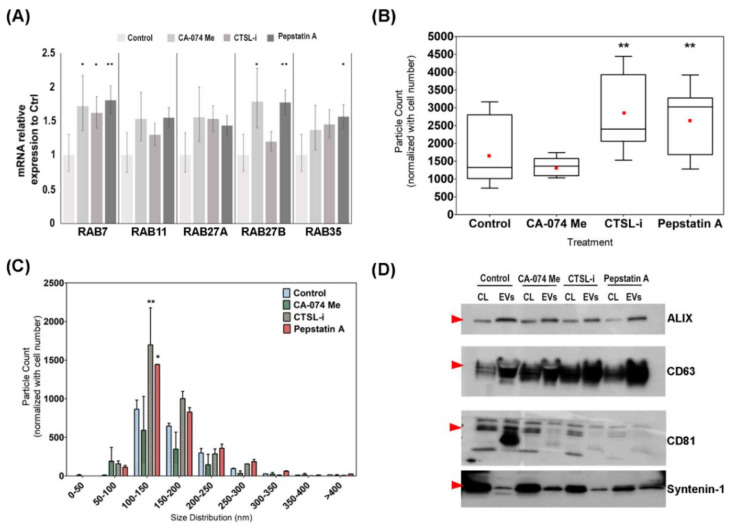
Chemical inhibition of cathepsin D modulate the EVs content. (**A**) Gene expression analysis of RAB GTPases after chemical inhibition of cathepsin proteolytic activities. mHypoA 2/28 cells were incubated in media containing 10 µM of CTSB inhibitor (CA-074 Me), 20 µM of CTSD inhibitor (pepstatin A (Pep-A)) or 10 µM CTSL inhibitor II (CTSL-i) for 24 h and collected for gene expression analysis of RAB proteins. β-actin was used as the reference gene and fold change was calculated using the ∆∆CT method. Error bars indicate S.E.M of three biological replicates (Student T-test, * *p* < 0.05; ** *p* < 0.01). (**B**) Box plots showing EVs concentration from control, CA-074 Me, CTSL-i and Pep-A treated mHypoA 2/28 cells as measured through nanoparticle tracking analysis (NTA). EVs were quantified by particle count that was normalized against total cell count for each condition. Red dot represents the mean particle count for each treatment. Error bars indicate S.E.M of three biological replicates (One-way ANOVA, ** *p* < 0.01). (**C**) Comparison of the size distribution of EVs from the various treatment, Bin size is 50 nm. Number of particles in each bin size is normalized against total cell count. Error bars indicate S.E.M of three biological replicates (Two-way ANOVA, * *p* < 0.05, ** *p* < 0.01). (**D**) Western blot analysis of known EVs marker ALIX, CD63, CD81 and syntenin-1 following 24 h of chemical inhibition against CTSB, CTSD, and CTSL in mHypoA2/28 cells. (CL: Cell lysate; EVs: Extracellular Vesicles; Red arrow indicate the band of interest.).

RAB GTPases regulate both ESCRT-dependent and ESCRT-independent EVs biogenesis and secretion [56]. RAB7 is involved in late endocytic vesicle trafficking [57] and vesicle secretion in a ESCRT-dependent [12] or independent manner [58]. While RAB11 regulates the movement of vesicles from recycling endosomes to the plasma membrane for exocytosis [59], RAB27a and RAB27b are involved in ESCRT-dependent EVs secretion [60]. Finally, Rab35 is involved in fast endosome recycling pathway [61] as well as tethering late endosome to the plasma membrane in an ESCRT-independent manner [14]. Therefore, gene expression analysis on these five Rab GTPases were conducted to understand whether cathepsin inhibition affects EVs secretion in mHypoA 2/28 cells. RT-qPCR analysis of cathepsin-inhibited mHypoA 2/28 cells revealed that CTSB inhibition increased the expression of *RAB7* and *RAB27b*; CTSL inhibition resulted in increased *RAB7* expression; and CTSD inhibition resulted in an increase of *RAB7*, *RAB27b* and *RAB35* expression (Figure 4A). These results suggested two things: (1) The increased in Rab GTPases expression upon cathepsin inhibition indicated increased EVs secretion. (2) Each cathepsin may regulate different subpopulation of vesicles for secretion.

Subsequently, nanoparticle tracking analysis (NTA) was utilized to determine whether the increased in Rab GTPases expression resulted in an actual increase in EVs secretion. A box and whisker plot were constructed and NTA measurement revealed that CTSL-i and Pep-A treatment on mHypoA 2/28 cells significantly increased EVs secretion (Figure 4B). 

The EVs particle count per non-treated mHypoA 2/28 cell had a mean value of 1657 and the lower quantile (Q1), median (Q2) and upper quantile (Q3) were 1012, 1326 and 2801 respectively, while the EVs particle count from CTSB-inhibited mHypoA 2/28 had similar value with a mean of 1352 and the Q1, Q2 and Q3 were 1096, 1363 and 1572. The EVs particle count was higher in the two other conditions and the mean, Q1, Q2 and Q3 value were 2890, 2060, 2402 and 3929 for the EVs derived from CTSL-inhibited mHypoA 2/28 cells and 2714, 1687, 3023 and 3277 for the EVs derived from CTSD-inhibited mHypoA 2/28 cells.

Further analysis was done to profile the size distribution of the EVs isolated from the cathepsin-inhibited mHypoA 2/28 cells and the higher particle count from CTSL and CTSD inhibited cells were attributed to the increased secretion of particles between the size of 100–150 nm (Figure 4C). Subsequently, Western blot analysis on known EVs markers such as ALIX, CD63, CD81 and syntenin-1 was conducted on cathepsin-inhibited mHypoA 2/28 EVs to examine if the treatment resulted in a change in EVs subpopulation (Figure 4D). Interestingly, when treated with either CTSL-I or Pep-A, mHypoA secreted EVs that are enriched in CD63, while inhibition of CTSB, CTSD or CTSL resulted in a decrease of CD81 expression in EVs. CTSD inhibition also resulted in a decreased in cellular CD81 expression. The EVs and cellular expression of ALIX and syntenin-1 remained largely unchanged after cathepsin inhibition.

## 4. Discussion

The exponential growth in EVs research in the last two decades highlighted their importance in the intercellular communication. Encapsulated by a lipid bilayer, the EVs act as protective carrier and transport a wide repertoire of biological molecules such as proteins, RNA, and lipids across the lymphatic and circulatory system and mediate biological responses at distant site [1,62]. Previously, our lab had also established the role of EVs in hypoxia-induced cancer progression, neurodegenerative disease and cardiovascular disease [3,26,63,64]. Despite the growing knowledge of EVs functions, the mechanism and regulation of their biogenesis and secretion remained poorly understood. Nonetheless, recent studies have shown the intricate link between lysosomal activities and EVs secretion. In this study, we established a pSILAC-based quantitative proteomic methodology for the study of EVs biogenesis. Through profiling of the newly synthesized proteome of the EVs, lysosome and total cell lysate from mHypoA 2/28 adult hypothalamus cell line, the spatial distribution of these newly synthesized proteins were analyzed and we demonstrated that newly synthesized protein that preferentially sorted into the EVs is involved in EVs biogenesis and secretion. Firstly, gene ontology analysis indicated that regulation of EVs biogenesis and secretion is an active process that required constant synthesis of the involved proteins such as ESCRT proteins CHMP2A, TSG101, VPS4A, VPS4B, HGS and ALIX; proteins involved in MVBs formation, syntenin-1 and syndecan; and RAB7A that is involved in exosome secretion were actively synthesized by the mHypoA 2/28 cells. This finding indicated that the application of pSILAC-based quantitative proteomics in the study of EVs biogenesis was appropriate.

Next, hierarchical cluster analysis on the three sub-proteome datasets identified a group of cathepsin proteins that were actively synthesized and sorted into the EVs of which CTSB and CTSD was subjected to further analysis to elucidate their role in EVs biogenesis and they was compared to CTSL, which has a higher protein synthesis ratio in the lysosome. Based on the data, CTSD may play a greater role in EVs biogenesis when compared with CTSB and CTSL. The reason being that CTSL is predominantly localized to the lysosome fraction, and pro-CTSL but not mature CTSL are enriched in the EVs. Pro-CTSL possessed minimal proteolytic activity and secreted pro-CTSL are only active in the presence of glycosaminoglycan at the extracellular matrix [65] indicating that the pro-CTSL in the EVs are not likely to be functionally active prior to secretion. The increase in EVs secretion and RAB7 gene expression from CTSL inhibition may be attributed to the perturbation of the lysosome status. Recent studies have shown that the inhibition of lysosome activities with chemical compound such as Bafilomycin A resulted in the perturbed cells relying on EVs secretion pathway as an alternative method for cellular waste disposal [66]. As an essential protease in the lysosomal system [67], the inhibition of CTSL likely resulted in dysregulation of lysosomal activity and hence the increase in EVs secretion could be a coping mechanism by the cells to remove accumulated cellular waste. On the other hand, CTSB inhibition in mHypoA 2/28 cells caused an increase in cellular *RAB7* and *RAB27B* gene expression but this did not translate into an increase in EVs secretion. The inhibition may have resulted in lysosomal dysregulation such as lysosome enlargement and accumulation of lysosome in the cytosol [68,69] rather than affecting the EVs secretion rate.

Lastly, our data indicated newly synthesized CTSD may play a possible role in EVs biogenesis. Firstly, mature CTSD are enriched in the endosome rather than the lysosome of the mHypoA 2/28 cells and the mHypoA 2/28 EVs contained only newly synthesized CTSD while pre-existing CTSD together with its newly synthesized counterpart are found in the lysosome instead. This suggested that actively synthesized CTSD are sorted into the EVs while the lysosome is the storage site for CTSD to perform housekeeping activities as such pre-existing CTSD proteins are found in the lysosome. Next, both pro-CTSD and mature CTSD were identified in the EVs samples and it is plausible that they may have differential role in EV biology. The matured CTSD is likely to be involved in EV biogenesis as indicated by our data, while the pro-CTSD may have other functional role upon vesicle uptake by the recipient cells through endocytosis. As the engulfed EVs travelled through the endo-lysosomal system of the recipient cell, the acidification of the endosome can result in the activation of the pro-CTSD into CTSD in the recipient cell to exert its cellular proteolytic functions.

Subsequently, we demonstrated that, chemical inhibition of CTSD activity increased the cellular gene expression of *RAB7*, *RAB27b* and *RAB35*. These RAB GTPases are involved in the trafficking of mature MVBs, which typically contain CD63, to the plasma membrane for EVs secretion [12,14,58,60]. In addition, enhanced secretion of CD63 and Rab35-containing EVs were associated with neurological disorder such as Down syndrome which was suggested as a mean for the cells to alleviate endosomal pathology through EVs secretion [70]. We also found that the increased RAB GTPases gene expression in our dataset coincide with enhanced secretion of EVs between the size of 100–150 nm upon CTSD inhibition. This observation in turn could be contributed by the presence of CD63-containing EVs as CTSD inhibition also altered the EVs composition by releasing more endosomal-associated CD63-containing EVs but reducing plasma membrane-associated CD81-containing EVs secretion as indicated by western blot analysis. Taken together, this would suggest that CTSD play a role in modulating the type of EVs to be secreted out into the extracellular milieu. Future study on the protein cargoes in the respective EVs subtypes would facilitate the understanding of the biological role of CTSD in EVs biology and cellular homeostasis. On the other hand, CTSB and CTSL inhibition were found to also affect the EVs expression of CD63 and CD81. This observation maybe due to the secondary effect mediated by the absence of CTSB and CTSL activities as both CTSB and CTSL were demonstrated to be involved in CTSD processing [71]. Therefore, CTSB and CTSL inhibition may have impeded the activity of CTSD.

The reduction in CD81^-^containing EVs from CTSD chemical inhibition suggest that CTSD may be involved in EVs biogenesis that is localized to the plasma membrane. EVs biogenesis along the plasma membrane is dependent on a lipid-based mechanism [19]. Several proteins that are involved in sphingolipid metabolism were also found in our EVs proteome dataset and this included prosaposin (PSAP), acid ceramidase (ASAH1) and acid sphingomyelinase (SMPD1) (Table S1). PSAP is a precursor to the sphingolipid activator proteins (saposins) that are involved in the hydrolysis of sphingolipid [72] and it binds and forms complexes with Pro-CTSD in the endoplasmic reticulum [73]. Under acidic condition, PSAP catalyzed the activation of pro-CTSD to CTSD and the activated CTSD in turn processed PSAP into saposin, with saposin D being the dominant form [74]. Saposin D acts as a cofactor in acid ceramidase (ASAH1)-mediated degradation of ceramide into sphingosine [75] and also in acid sphingomyelinase(SMPD1)-mediated hydrolysis of sphingomyelin into ceramide [76]. Furthermore, SMPD1-derived ceramide is an intracellular binding partner of CTSD and enhances the proteolytic activity of CTSD [77]. Therefore, it is plausible that the association of CTSD with PSAP, ASAH1 and SMPD1 could regulate sphingolipid metabolism related to EVs biogenesis.

Given that neurons are terminally differentiated, they are particularly sensitive to stress from lysosome dysregulation and cellular waste accumulation. This is evident in neurological disease such as Alzheimer’s and Parkinson disease. Future studies should seek to understand the functions of CTSD in sphingolipid metabolism during EVs biogenesis in both physiological and pathology settings as this would help to delineate CTSD-mediated EVs functions toward cellular homeostasis and lysosomal status. On the other hand, the mHypoA 2/28 cell originated from the pro-opiomelanocortin (POMC) neuron that is a gateway between the nervous system and the endocrine system, therefore mHypoA 2/28-derived EVs may have a prospective role in regulating energy homeostasis and stress. It would also be valuable to study the functional role of CTSD-regulated EVs secretion pertaining to hypothalamic stress due to over-nutrition, energy homeostasis regulation and stress response.

## Figures and Tables

**Table 1:** Gene ontology analysis identified newly synthesized cell lysate (**A**) and EVs (**B**) proteins that belong to the GO:1903543~positive regulation of exosomal secretion biological process.

**(A)**			
**Cell Lysate**			
**Gene Name**	**Protein** **Name**	**H/L Ratio**	**Unique Peptide**
CHMP2A	Charged multivesicular body protein 2A	5.67	5
HGS	Hepatocyte growth factor-regulated tyrosine kinase substrate	1.84	12
PDCD6IP	Alix	1.61	48
RAB7A	Ras-related protein RAB-7a	1.55	19
SDC1	Syndecan 1	100	3
SDCBP	Syntenin-1	24.62	5
TSG101	Tumor susceptibility gene 101	1.51	10
VPS4A	Vacuolar protein sorting-associated protein 4A	3.2	5
VPS4B	Vacuolar protein sorting-associated protein 4B	2.13	7
**(B)**			
**EVs**			
**Gene Name**	**Protein** **Name**	**H/L Ratio**	**Unique Peptide**
PDCD6IP	ALIX	1.64	21
RAB7A	Ras-related protein RAB-7a	5.64	5
SDCBP	Syntenin-1	2.73	7

**Table 2:** H/L ratio analysis of the ESCRT machinery. The ESCRT system consists of 4 protein complexes: ESCRT-0 (**A**), I (**B**), II (**C**), III (**D**), and the accessory proteins (**E**).

**(A)**							
**ESCRT-0**							
		**EVs**		**Lysosome**		**Cell Lysate**	
**Gene Name**	**Protein Name**	**H/L Ratio**	**Unique Peptide**	**H/L Ratio**	**Unique Peptide**	**H/L Ratio**	**Unique Peptide**
HGS	Hepatocyte growth factor-regulated tyrosine kinase substrate	-	-	100	2	1.81	12
STAM1	Signal transducing adapter molecule 1	-	-	-	-	1.45	13
STAM2	Signal transducing adapter molecule 2	-	-	-	-	2.67	12
**(B)**							
**ESCRT-I**							
		**EVs**		**Lysosome**		**Cell Lysate**	
**Gene Name**	**Protein Name**	**H/L Ratio**	**Unique Peptide**	**H/L Ratio**	**Unique Peptide**	**H/L Ratio**	**Unique Peptide**
MVB12A	Multivesicular body subunit 12A	0.01	1	-	-	2.93	5
MVB12B	Multivesicular body subunit 12B	-	-	-	-	7.76	3
TSG101 *	Tumor susceptibility gene 101	3.61	1	2.47	5	1.51	10
UBAP1	Ubiquitin-associated protein 1	-	-	-	-	1.60	11
VPS28	Vacuolar protein sorting-associated protein 28	2.61	3	-	-	1.25	11
VPS37A	Vacuolar protein sorting-associated protein 37A	-	-	-	-	2.03	7
VPS37B	Vacuolar protein sorting-associated protein 37B	-	-	-	-	3.40	5
VPS37C	Vacuolar protein sorting-associated protein 37C	-	-	-	-	2.19	6
**(C)**							
**ESCRT-II**							
		**EVs**		**Lysosome**		**Cell Lysate**	
**Gene Name**	**Protein** **Name**	**H/L Ratio**	**Unique Peptide**	**H/L Ratio**	**Unique Peptide**	**H/L Ratio**	**Unique Peptide**
VPS25	Vacuolar protein sorting-associated protein 25	-	-	-	-	0.70	9
VPS36	Vacuolar protein sorting-associated protein 36	-	-	-	-	2.84	12
**(D)**							
**ESCRT-III**							
		**EVs**		**Lysosome**		**Cell Lysate**	
**Gene Name**	**Protein Name**	**H/L Ratio**	**Unique Peptide**	**H/L Ratio**	**Unique Peptide**	**H/L Ratio**	**Unique Peptide**
CHMP1A	Charged multivesicular body protein 1A	-	-	-	-	0.01	2
CHMP1B	Charged multivesicular body protein 1B	-	-	-	-	2.11	1
CHMP2A	Charged multivesicular body protein 2A	-	-	-	-	5.67	5
CHMP2B	Charged multivesicular body protein 2B	-	-	-	-	1.18	3
CHMP3	Charged multivesicular body protein 3	-	-	-	-	2	4
CHMP4B	Charged multivesicular body protein 4B	2.15	1	100	1	1.89	5
CHMP4C	Charged multivesicular body protein 4C	-	-	-	-	21.31	4
CHMP5	Charged multivesicular body protein 5	-	-	-	-	2.14	7
CHMP6	Charged multivesicular body protein 6	-	-	-	-	1.98	3
CHMP7	Charged multivesicular body protein 7	-	-	-	-	16.08	2
IST1	IST1 homolog	-	-	0.48	2	1.59	8
**(E)**							
**ESCRT-Accessory Proteins**							
		**EVs**		**Lysosome**		**Cell Lysate**	
Gene Name	Protein Name	**H/L Ratio**	**Unique Peptide**	**H/L Ratio**	**Unique Peptide**	**H/L Ratio**	**Unique Peptide**
PDCD6IP	ALIX	5.64	21	1.93	23	1.61	48
VPS4A	Vacuolar protein sorting-associated protein 4A	-	-	-	-	3.2	5
VPS4B	Vacuolar protein sorting-associated protein 4B	-	-	9.86	2	2.13	7
VTA1	Vacuolar protein sorting-associated protein VTA1 homolog	-	-	8.00	4	1.18	7

* EV’s TSG101 is found in one biological replicate only.

**Table 3:** H//L analysis of ESCRT dependent and independent mechanism for MVBs formation. The ALIX-syntenin-syndecan (**A**), tetraspanins-enriched domain (**B**) and ceramide-based EVs biogenesis (**C**) are alternate mode of MVBs formation.

**(A)**							
**ALIX-Syntenin-Syndecan Axis**							
		**EVs**		**Lysosome**		**Cell Lysate**	
**Gene Name**	**Protein Name**	**H/L Ratio**	**Unique Peptide**	**H/L Ratio**	**Unique Peptide**	**H/L Ratio**	**Unique Peptide**
PDCD6IP	ALIX	5.64	21	1.93	23	1.61	48
SSDCBP	Syntenin-1	2.73	7	10.8	7	24.62	5
SDC1	Syndecan 1	-	-	-	-	100	3
**(B)**							
**Tetraspanin-enriched Domain**							
		**EVs**		**Lysosome**		**Cell Lysate**	
**Gene Name**	**Protein Name**	**H/L Ratio**	**Unique Peptide**	**H/L Ratio**	**Unique Peptide**	**H/L Ratio**	**Unique Peptide**
CD9	CD9	2.40	2	3.41	3	2.13	4
CD63	CD63	0.19	1	0.73	5	1.53	4
CD81	CD81	10.34	3	30.61	2	7.43	3
**(C)**							
**Ceramide-based EVs Biogenesis**							
		**EVs**		**Lysosome**		**Cell Lysate**	
**Gene Name**	**Protein Name**	**H/L Ratio**	**Unique Peptide**	**H/L Ratio**	**Unique Peptide**	**H/L Ratio**	**Unique Peptide**
SMPD1	Sphingomyelin phosphodiesterase	0.01	1	-	-	16.24	1
SMPD2	Sphingomyelin phosphodiesterase 2	-	-	-	-	1.56	7
SMPD3	Sphingomyelin phosphodiesterase 3	-	-	-	-	0.88	1

## Supplementary Materials

The following are available online at https://www.mdpi.com/2073-4409/9/5/1320/s1, Figure S1: Number of proteins identified in Exocarta (**A**) and Vesiclepedia (**B**). Figure S2: pSILAC-based quantitative proteomics analysis of mHypoA 2/28 cell lysate, lysosome and EVs. Table S1: List of proteins that are associated with sphingolipid metabolism.
