# Supplementary material for: Exploring Extracellular Vesicles Biogenesis in Hypothalamic Cells through a Heavy Isotope Pulse/Trace Proteomic Approach
Source: Cells. 2020 May 25;9(5):1320. doi: 10.3390/cells9051320 (PMC7291124; doi:10.3390/cells9051320)
Supplement: Supplementary file 1 [file cells-09-01320-s001.zip › Supplementary Figure.pdf]

# Supplementary Figure

**A**

| Proteins quantified in the pSILAC dataset that matches the top 25 proteins in Exocarta | | | | |
|---|---|---|---|---|
| ACTB | ACTG1 | ALB | ALDOA | ANXA2 |
| ANXA5 | CD63 | CD81 | CD9 | CFL1 |
| EEF1A1 | EEF2 | ENO1 | GAPDH | HSP90AA1 |
| HSP90AB1 | HSPA8 | LDHA | MSN | PDCD6IP |
| PGK1 | SDCBP | YWHAE | YWHAZ | |

**B**

| Proteins quantified in the pSILAC dataset that matches the top 100 EV protein in Vesiclepedia | | | | |
|---|---|---|---|---|
| A2M | ACLY | ACTB | ACTN1 | ACTN4 |
| AHCY | ALB | ALDOA | ANXA1 | ANXA2 |
| ANXA5 | C3 | CCT2 | CCT3 | CCT4 |
| CCT6A | CD63 | CD81 | CD9 | CDC42 |
| CFL1 | CLIC1 | CLTC | EEF1A1 | EEF2 |
| EIF4A1 | ENO1 | FASN | FLNA | FLOT1 |
| FN1 | GAPDH | GNAI2 | GNAS | GNB1 |
| GNB2 | GSN | HIST1H4A | HSP90AA1 | HSP90AB1 |
| HSPA8 | ITGB1 | KPNB1 | KRT1 | KRT10 |
| LDHA | LDHB | LGALS3BP | MYH9 | PDCD6IP |
| PFN1 | PGK1 | PKM | PRDX1 | RAB5C |
| RAB7A | RAC1 | RALA | RAN | RAP1B |
| SDCBP | TCP1 | TFRC | TLN1 | TPI1 |
| TSG101 | TUBB4B | UBA1 | VCP | YWHAE |
| YWHAG | YWHAQ | YWHAZ | | |

**Figure S1:** Number of proteins identified in Exocarta and Vesiclepedia

(A)

| | Identified Proteins | Quantified Proteins |
|---|---|---|
| Cell Lysate | 7327 | 6062 |
| Lysosome | 1545 | 1125 |
| EVs | 545 | 392 |

(B)

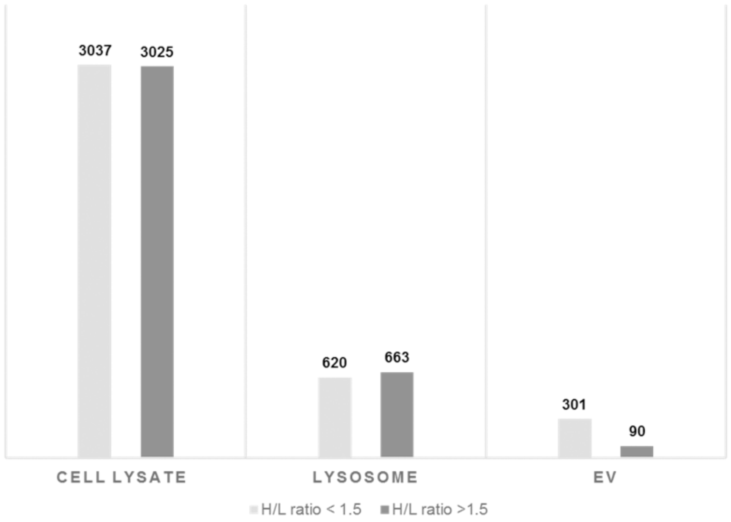


(C) (D) (E)

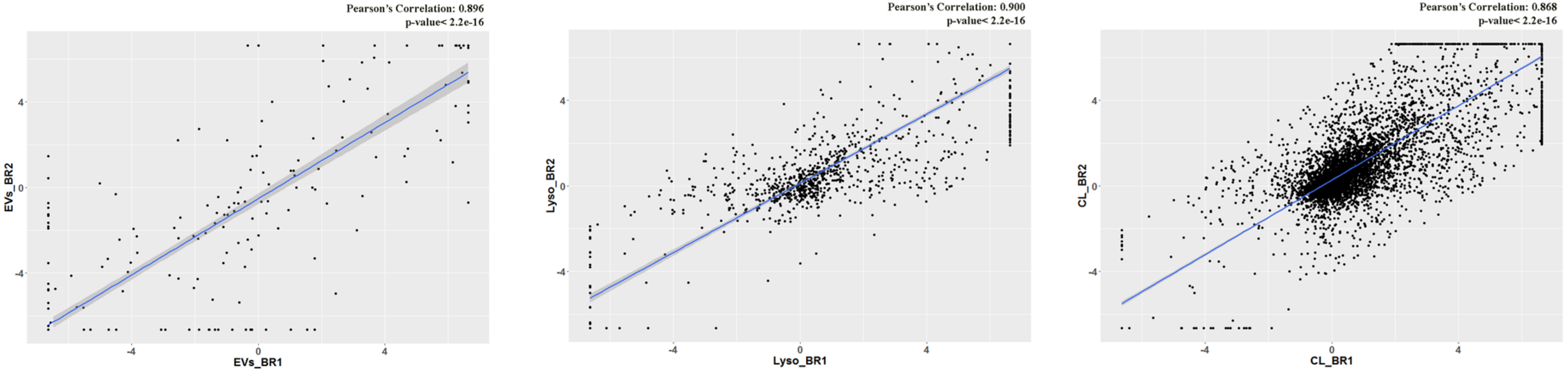


**Figure S2: pSILAC-based quantitative proteomics analysis of mHypoA 2/28 cell lysate, lysosome and EVs.** (A) Summary of proteins identified in the mHypoA cell lysate, lysosome and EV proteome respectively. Proteins were considered as quantified if they were identified in both biological replicates and proteins will large variation was omitted. (B) The quantified proteins were further subcategorized to newly syntheiszed protein (H/L ratio > 1.5) or pre-existing protein (H/L ratio< 1.5). (C-E) Pearson's correlation coefficients between two biological replicates from the EV, lysosome and cell lysate proteomes.
