# Supplementary material for: Exploring Extracellular Vesicles Biogenesis in Hypothalamic Cells through a Heavy Isotope Pulse/Trace Proteomic Approach
Source: Cells. 2020 May 25;9(5):1320. doi: 10.3390/cells9051320 (PMC7291124; doi:10.3390/cells9051320)
Supplement: Supplementary file 1 [file cells-09-01320-s001.zip › Table S1.docx]

Table S1: List of proteins that are associated with Ctsd interactions and activities.

|  | | | | | | | |
| --- | --- | --- | --- | --- | --- | --- | --- |
|  |  | **EVs** | | **Lysosome** | | **Cell lysate** | |
| **Gene Name** | **Protein Name** | **H/L ratio** | **Unique peptide** | **H/L ratio** | **Unique peptide** | **H/L ratio** | **Unique peptide** |
| PSAP | Prosaposin | 6.54 | 7 | 0.68 | 7 | -0.21 | 20 |
| ASAH1 | Acid ceramidase | 6.64* | 2 | 1.08 | 8 | -0.48 | 15 |
| SMPD1 | Acid sphingomyelinase | -6.64 | 1 | - | - | 4.02 | 1 |
| IGF2R | Cation-independent mannose 6-phosphate receptor | -5.67 | 10 | 6.64 | 14 | 2.90 | 54 |
| M6PR | Cation-dependent mannose 6-phosphate receptor | - | - | 0.47 | 3 | 0.14 | 5 |

** ASAH1 proteins was found in one of the two biological replicates in the EVs proteome.*
